# Proteomics of intracellular freezing survival

**DOI:** 10.1371/journal.pone.0233048

**Published:** 2020-05-26

**Authors:** Michael A. S. Thorne, Nina Kočevar Britovšek, Liam Hawkins, Kathryn S. Lilley, Kenneth Storey

**Affiliations:** 1 British Antarctic Survey, Cambridge, United Kingdom; 2 Cambridge Centre for Proteomics, University of Cambridge, Cambridge, United Kingdom; 3 Biochemistry Department, Carleton University, Ottawa, Canada; Trent University, CANADA

## Abstract

*Panagrolaimus* sp. DAW1, a nematode cultured from the Antarctic, has the extraordinary physiological ability to survive total intracellular freezing throughout all of its compartments. While a few other organisms, all nematodes, have subsequently also been found to survive freezing in this manner, *P*. sp. DAW1 has so far shown the highest survival rates. In addition, *P*. sp. DAW1 is also, depending on the rate or extent of freezing, able to undergo cryoprotective dehydration. In this study, the proteome of *P*. sp DAW1 is explored, highlighting a number of differentially expressed proteins and pathways that occur when the nematodes undergo intracellular freezing. Among the strongest signals after being frozen is an upregulation of proteases and the downregulation of cytoskeletal and antioxidant activity, the latter possibly accumulated before freezing much in the way the sugar trehalose has been shown to be stored during acclimation.

## Introduction

The Antarctic nematode, *Panagrolaimus* sp. DAW1 (previously called *Panagrolaimus davidi* and *Panagrolaimus* sp. CB1) [1,2], is the best understood organism able to survive the extreme disruption of intracellular freezing throughout all of its compartments [3,4,5]. In addition, under a slow freezing regime, the nematode is able to cryoprotectively dehydrate [6]. As part of an ongoing effort to understand the molecular mechanisms underlying this nematode’s extraordinary adaptations, both the transcriptome and the genome have been sequenced [7], RNA expression profiling during intracellular freezing has been investigated [8], and the potential for functional genomic methods has been explored [9]. The current paper, a whole proteomic analysis of *P*. sp DAW1 when intracellularly frozen, continues and extends this effort.

Much current and past theory (for example [10,11,12]) about freeze tolerance and cell behaviour under freezing regimes may not hold in the case of *P*. sp. DAW1, since the very assumption that lies at the heart of most of these theories, that intracellular freezing is lethal, does not hold. While there are optimal conditions that allow *P*. sp. DAW1 to survive, such as not being under hypo- or hyper-osmotic stress [13], and ensuring a healthy nutritional status [14] (optimal conditions of the pattern and distribution of the formed ice [15] also has an effect on survival rate), once these are met, a culture of *P*. sp DAW1 that has gone through a freeze-thaw cycle has survival rates of around 80% (see [8]), and is able to produce progeny.

Very little is presently understood in terms of the molecular details of how an organism is able to survive intracellular freezing (in all, rather than in select, cells or compartments such as with *Eurosta solidaginis* [16]), and there is much to be learnt in order to build up a coherent picture. Yet despite the unique nature of this nematode’s cold-tolerance mechanism, it has not come as much surprise that, so far, many of the same proteins and pathways involved are also implicated in other cold tolerant and cold avoiding systems and cover a diverse range of functions [7,8]. These include trehalose (see also [17]), late embryogenic abundant (LEA) proteins, aquaporins, and reactive oxygen species (ROS) related genes. One unexpected gene that has shown a very strong upregulated signal during freezing was a neprilysin-like zinc metalloprotease [8], and understanding its function in this context is of high priority.

However, despite one report that has subsequently proven inconclusive [18], to date there has been no success in finding any ice binding or ice active proteins, important for example for recrystallization inhibition [19], vital for preventing damage to the membrane during thawing. Finding any clues as to how such ice active proteins function, what pathways they function within, what signals they respond to, and most importantly what they are, remains a key goal in studying this extraordinary nematode.

## Methods & materials

### *Panagrolaimus* sp. DAW1 protein extraction

Nematode samples from two intracellular freezing stages (short term freezing: rapid descent from +5ºC to -10ºC and then ice nucleated; and long term freezing: rapid descent from +5ºC to -10ºC, ice nucleated and then held at -10ºC for 24 h) and a control stage (acclimated at +5ºC for three days after being brought down from culture growth conditions at +20ºC) were described in detail in [7,8]. Replicate (3) samples were cut to approximately 100 mg and homogenized with a pestle after the addition of 500 μl lysis buffer (50 mM HEPES pH 7.8/0.1% SDS supplemented with protease inhibitors—cOmplete^™^, Mini Protease Inhibitor Cocktail from Roche). They were then vortexed, sonicated on ice for 5 min, and incubated on ice for approximately 30 min. Finally, they were centrifuged twice (16000 × g at 4°C for 15 min and 5 min, respectively) and protein concentration was measured with the Pierce BCA (bicinchoninic acid) Protein Assay Kit according to the manufacturer’s instructions.

### In gel digestion and mass spectrometry

20 ug proteins were prepared in Laemmli buffer, reduced with 50 mM DTT 10 min at 75°C, alkylated with 55 mM IAA 30 min at RT in the dark and loaded on 12% pre-cast gels (Bio-Rad). After SDS-PAGE, gels were fixed 45 min, stained with Coomassie Brilliant Blue for 2 h and de-stained with water 3 x 30 min. Each gel lane was cut in 5 bands (see S1 Fig) that were further cut in 1 mm^2^ pieces, de-stained and digested with trypsin (ratio 1:20) at 37°C overnight. The supernatant was then collected and two more extraction steps were performed on the remaining gel pieces using 50% acetonitrile (ACN)/5% formic acid (FA) and incubated for 15 min at 37°C. The liquid from successive extractions was pooled and then freeze-dried. After lyophilisation, peptides were re-suspended in 20 μl 3% ACN/0.1% FA. 13 μl for band 1 and 15 μl for bands 2–5 was loaded on QExactive for 1h runs. All LC-MS/MS experiments were performed using a Dionex Ultimate 3000 RSLC nanoUPLC (Thermo Fisher Scientific Inc, Waltham, MA, USA) system and a QExactive Orbitrap mass spectrometer (Thermo Fisher Scientific Inc, Waltham, MA, USA) as described recently [20]. The mass spectrometry proteomics data have been deposited to the ProteomeXchange Consortium via the PRIDE [21] partner repository with the dataset identifier PXD018121.

### Proteomic data analysis

The data was processed with MaxQuant v1.6.0.1 [22], using the default parameters unless stated otherwise. Raw files were searched against a database generated from a six-frame protein translation of the published transcriptome [7] including common contaminants. Carbamidomethyl (C) was set as fixed modification, and oxidation (M) and deamidation (NQ) were set as dynamic modifications. Up to two missed cleavages were allowed and the FDR was set to 1%. “Match between runs” was enabled, normalised *LFQ Intensity* [23] was used for quantification of the summed up extracted ion current intensities and selection was also based on the normalization (LFQ) with the LFQ minimum ratio count set to 1.

### Differential expression analysis

Contaminants and reverse hits were removed from the dataset. Proteins were filtered for those with non-zero maxLFQ intensities in 2 of 3 replicates in all conditions, resulting in 1844 (76%) unique protein hits. ProStaR [24] was used for statistical analysis. The K-nearest neighbor algorithm was used for data imputation, and ProStaR’s FDR calibration tools and the Limma package [25] were used to determine significant differences between control and experimental conditions (*p* < 0.05). Differential expression was visualized with volcano plots (see S2 and S3 Figs) using a custom python script and Matplotlib [26], where thresholds are set to ±2-fold change and *p* < 0.05. Proteins that meet these criteria and are up-regulated between control and experimental conditions are blue (and listed in Tables 1 & 2) and those down-regulated are red (and listed in S1 & S2 Tables).

**Table 1:** Proteins upregulated after immediate freezing at -10ºC and ice nucleated compared to control. Symbols: * match shared between both treatments (-10ºC and -10º for 24h); # annotation determined only through motif, potentially contaminant; § indicates the match is part of a small STRING network (see Fig 1).

Match	Annotation	Log2FC
PdU009721_v1.1_crick_0§	CBR-CYN-3 protein (immunosuppressant)	3.65
PdU037792_v1.1_crick_1*§	60S ribosomal protein L10a	3.02
PdU041703_v1.1_crick_2*	60S ribosomal protein L27a-like	2.78
PdU056397_v1.1_crick_2	Ras-like GTP-binding protein rhoA	2.39
PdU055796_v1.1_watson_0	CRE-CLEC-51 protein	2.07
PdU056258_v1.1_crick_2§	Cytokinesis, Apoptosis, RNA-associated family member (car-1)	1.96
PdU058721_v1.1_crick_2	MD-2-related lipid-recognition domain-containing protein	1.88
PdU058717_v1.1_crick_1*#	pfam04147, Nop14, Nop14-like family.	1.62
PdU057565_v1.1_crick_0	perm-4 –sugar modifying enzyme	1.61
PdU000126_v1.1_crick_0	Eukaryotic translation initiation factor 3 subunit A	1.60
PdU059047_v1.1_crick_1	Serine/threonine-protein phosphatase 4 catalytic subunit 1	1.54
PdU020997_v1.1_crick_0	6-pyruvoyl tetrahydrobiopterin synthase, partial	1.51
PdU056518_v1.1_crick_2*	dhs-23 Dehydrogenases, Short chain family member	1.50
PdU055863_v1.1_crick_1*§	ML domain containing protein	1.48
PdU014169_v1.1_crick_1*§	C. briggsae CBR-RPB-7 protein	1.46
PdU060431_v1.1_crick_0	nuclear export mediator factor NEMF homolog	1.45
PdU002121_v1.1_crick_0§	Crooked neck-like protein 1	1.42
PdU054484_v1.1_crick_2*§	calcium activated nucleotidase 1	1.41
PdU010120_v1.1_crick_2*§	heterogeneous ribonuclear particle protein	1.40
PdU010249_v1.1_crick_1*	Eukaryotic translation initiation factor 4H	1.38
PdU053765_v1.1_crick_1§	nath-10 RNA cytidine acetyltransferase	1.37
PdU058254_v1.1_crick_1§	Ras-related protein Rab-1A	1.36
PdU058887_v1.1_crick_0§	CBN-NST-1 protein	1.34
PdU008151_v1.1_crick_2*	ubxn-4 UBX domain-containing protein 4	1.34
PdU054635_v1.1_crick_0#	Fibrillarin	1.34
PdU003448_v1.1_crick_1	Inositol-3-phosphate synthase	1.34
PdU057628_v1.1_crick_0§	Transthyretin-like protein 5	1.31
PdU013526_v1.1_crick_0	activator of Hsp90 ATPase	1.28
PdU016560_v1.1_crick_1#	pfam09770, PAT1, Topoisomerase II-associated protein PAT1.	1.27
PdU007626_v1.1_crick_2*§	Eukaryotic translation initiation factor 3 subunit F	1.25
PdU002606_v1.1_crick_0	similar to Protein-kinase, interferon-inducible double stranded RNA dependent inhibitor (P58 repressor)	1.19
PdU056065_v1.1_crick_1*§	CaM Kinase Kinase family member (ckk-1)	1.18
PdU005100_v1.1_crick_0§	C. briggsae CBR-VPS-26 protein	1.17
PdU013332_v1.1_crick_2	Dipeptidyl peptidase 3, partial	1.16
PdU055293_v1.1_crick_0	DnaJ domain-containing protein	1.16
PdU013338_v1.1_crick_0*	SEC-2 protein	1.15
PdU059396_v1.1_crick_0§	ATP-dependent RNA helicase DDX55	1.15
PdU058158_v1.1_crick_1	mpst-1 Putative thiosulfate sulfurtransferase	1.13
PdU011528_v1.1_crick_1	Steroidogenic acute regulatory-like protein 1	1.10
PdU012850_v1.1_crick_0	Alcohol Dehydrogenase Class-III	1.09
PdU003565_v1.1_crick_2*	ZU5 and Death domain containing protein	1.08
PdU003464_v1.1_crick_2*§	Cytokinesis, Apoptosis, RNA-associated family member (car-1)	1.07
PdU000244_v1.1_crick_0§	Ankyrin repeat and FYVE domain-containing protein 1	1.03
PdU055430_v1.1_crick_1	variant SH3 domain-containing protein	1.03
PdU019049_v1.1_crick_0*#	ND5, NADH dehydrogenase subunit 5	1.02
PdU000449_v1.1_crick_0§	HBS1-like protein, partial	1.01
PdU006224_v1.1_crick_1	myosin heavy chain, nonmuscle type 1	1.01

**Table 2:** Proteins upregulated after being frozen at -10ºC and held for 24 h compared to control. Symbols as described in Table 1, although § indicates match part of a small STRING network as depicted in Fig 2. Matches specifying “-” indicate where no annotation was resolved and are potentially contaminants.

Match	Annotation	Log2FC
PdU003252_v1.1_crick_1	U4/U6 small nuclear ribonucleoprotein hPrp4	3.64
PdU055730_v1.1_crick_2	intercellular adhesion molecule 2 precursor variant	2.84
PdU041703_v1.1_crick_2*	60S ribosomal protein L27a-like	2.50
PdU059409_v1.1_crick_2§	GTP-binding nuclear protein Ran	2.42
PdU021494_v1.1_crick_0	Serine/threonine-protein phosphatase PP1 isozyme 2	2.20
PdU037792_v1.1_crick_1*§	60S ribosomal protein L10a	2.11
PdU059328_v1.1_crick_2	Glycogenin-1	1.87
PdU017506_v1.1_crick_2	CRE-LEA-1 protein	1.83
PdU054484_v1.1_crick_2*	calcium activated nucleotidase 1	1.73
PdU005138_v1.1_crick_1	Septin-8-A	1.70
PdU010120_v1.1_crick_2*§	heterogeneous ribonuclear particle protein	1.62
PdU019049_v1.1_crick_0*#	ND5, NADH dehydrogenase subunit 5	1.62
PdU014335_v1.1_crick_2	soc-2 Leucine-rich repeat protein	1.57
PdU055863_v1.1_crick_1*	ML domain containing protein	1.56
PdU058717_v1.1_crick_1*#	pfam04147, Nop14, Nop14-like family.	1.49
PdU010661_v1.1_crick_1	Lipid Binding Protein family member (lbp-3)	1.47
PdU012701_v1.1_crick_2	-	1.46
PdU003565_v1.1_crick_2*	ZU5 and Death domain containing protein	1.43
PdU055365_v1.1_crick_1	-	1.42
PdU004216_v1.1_crick_0	ADP-dependent glucokinase	1.42
PdU014738_v1.1_crick_2	Signal peptidase complex subunit 3	1.38
PdU006031_v1.1_crick_2	Nuclear movement protein	1.38
PdU056616_v1.1_crick_0	tufm-1 Elongation factor Tu	1.36
PdU054334_v1.1_crick_1	FXNA Endoplasmic Reticulum Metallopeptidase	1.36
PdU008847_v1.1_crick_1	putative-ribose 5-phosphate isomerase	1.35
PdU059718_v1.1_crick_1	-	1.35
PdU021476_v1.1_crick_2	Ral GTPase-activating protein subunit beta	1.34
PdU056597_v1.1_crick_2	speckle-type POZ protein-like	1.32
PdU019111_v1.1_crick_1	-	1.28
PdU001096_v1.1_crick_1§	Aminopeptidase N	1.28
PdU013338_v1.1_crick_0*	SEC-2 protein	1.26
PdU019994_v1.1_crick_0	b-cell receptor-associated protein 31-like protein	1.22
PdU055636_v1.1_crick_1	2,3-bisphosphoglycerate-independent phosphoglycerate mutase	1.22
PdU010249_v1.1_crick_1*	Eukaryotic translation initiation factor 4H	1.21
PdU036532_v1.1_crick_0§	rpt-6 Proteasome Regulatory Particle, ATPase-like family member	1.20
PdU056065_v1.1_crick_1*	CaM Kinase Kinase family member (ckk-1)	1.18
PdU020339_v1.1_crick_0	Aromatic-L-amino-acid decarboxylase	1.16
PdU000959_v1.1_crick_2	-	1.15
PdU007626_v1.1_crick_2*§	Eukaryotic translation initiation factor 3 subunit F	1.14
PdU004484_v1.1_crick_2	Protein farnesyltransferase subunit beta	1.14
PdU053876_v1.1_crick_1§	rpoa-1 DNA-directed RNA polymerase	1.14
PdU017441_v1.1_crick_2§	Neprilysin-1	1.12
PdU056518_v1.1_crick_2*	dhs-23 Dehydrogenases, Short chain family member	1.10
PdU056368_v1.1_crick_0	Kelch repeat type 1 domain containing protein	1.10
PdU014169_v1.1_crick_1*§	C. briggsae CBR-RPB-7 protein	1.09
PdU021904_v1.1_crick_1	oxidoreductase, short chain dehydrogenase/reductase family protein	1.07
PdU013171_v1.1_crick_2§	Nascent polypeptide-associated complex subunit alpha	1.06
PdU003464_v1.1_crick_2*§	Cytokinesis, Apoptosis, RNA-associated family member (car-1)	1.05
PdU005496_v1.1_crick_2	seld-1 Probable selenide, water dikinase	1.05
PdU008151_v1.1_crick_2*	ubxn-4 UBX domain-containing protein 4	1.03
PdU007056_v1.1_crick_1	Spartin	1.02
PdU058251_v1.1_crick_2	putative adenylyl cyclase CyaB	1.02
PdU057456_v1.1_crick_2	CBN-EMB-8 protein	1.02
PdU012449_v1.1_crick_2§	Cytochrome c oxidase subunit 6A	1.00

### Gene set enrichment and network analysis

For determining gene ontology required for gene set enrichment analysis (GSEA) in addition to annotating the differentially expressed matches, the *P*. sp. DAW1 proteome was aligned using BLASTP [27] with the most closely related species with high GO term [28] annotation coverage, *C*. *elegans*. InterProScan [29] was used to enhance downstream functional annotation. Both BLASTP and InterProScan results were then used to annotate the proteome with GO terms using Blast2GO [30]. The GOstats R package [31] was used to determine enrichment of the GO terms among the treatments, with both up- and down-regulated terms in each treatment brought together for a broader picture of the changes and the criteria for incorporation being the p-value threshold. The enriched Gostats, describing the Biological Processes (BP), Cellular Components (CC), and Molecular Functions (MF), are shown in S3 (short term freezing) and S4 (long term freezing) Tables. REVIGO [32], which summarizes and creates networks of GO terms using semantic similarity, was used to summarize and visualize the GO term enrichment between control and experimental conditions. Results from GOstats were supplied to the REVIGO webserver and the *C*. *elegans* GO term database was used for GO term sizes. Enriched GO term networks from REVIGO were then visualized with Cytoscape [33] (S4 to S9 Figs) with an alternative visualisation as tree maps (see S10 to S15 Figs).

**Fig 1:**
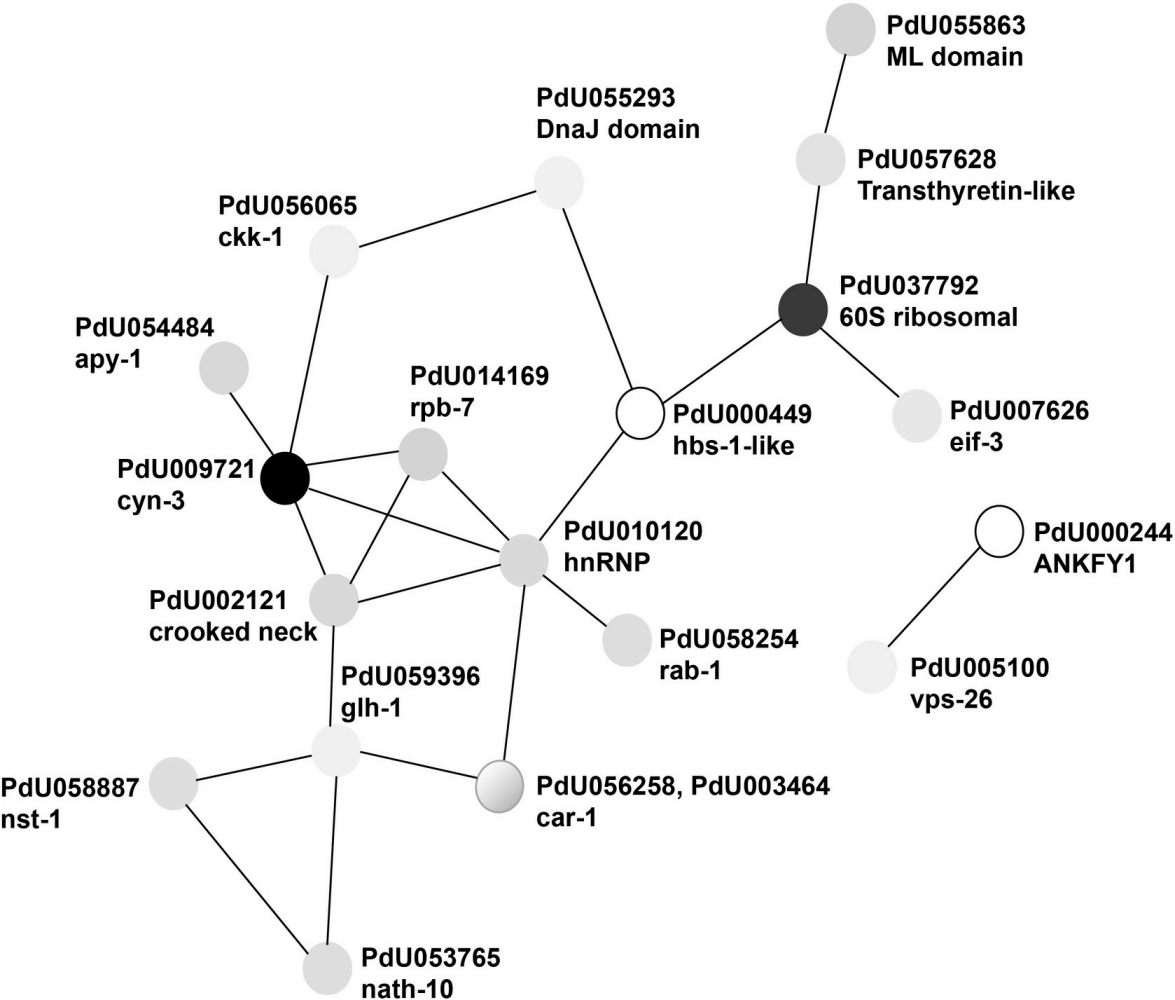
Depiction of the up-regulated short-term freezing protein matches that were grouped together by STRING [34] into a functional network. Matches from Table 1 that were disconnected from any other proteins were not depicted. Shading of the nodes is representative of the relative log fold change of the respective protein from Table 1. PdU056258 and PdU003464, represented as one node, has a shading reflecting the different log fold values.

**Fig 2:**
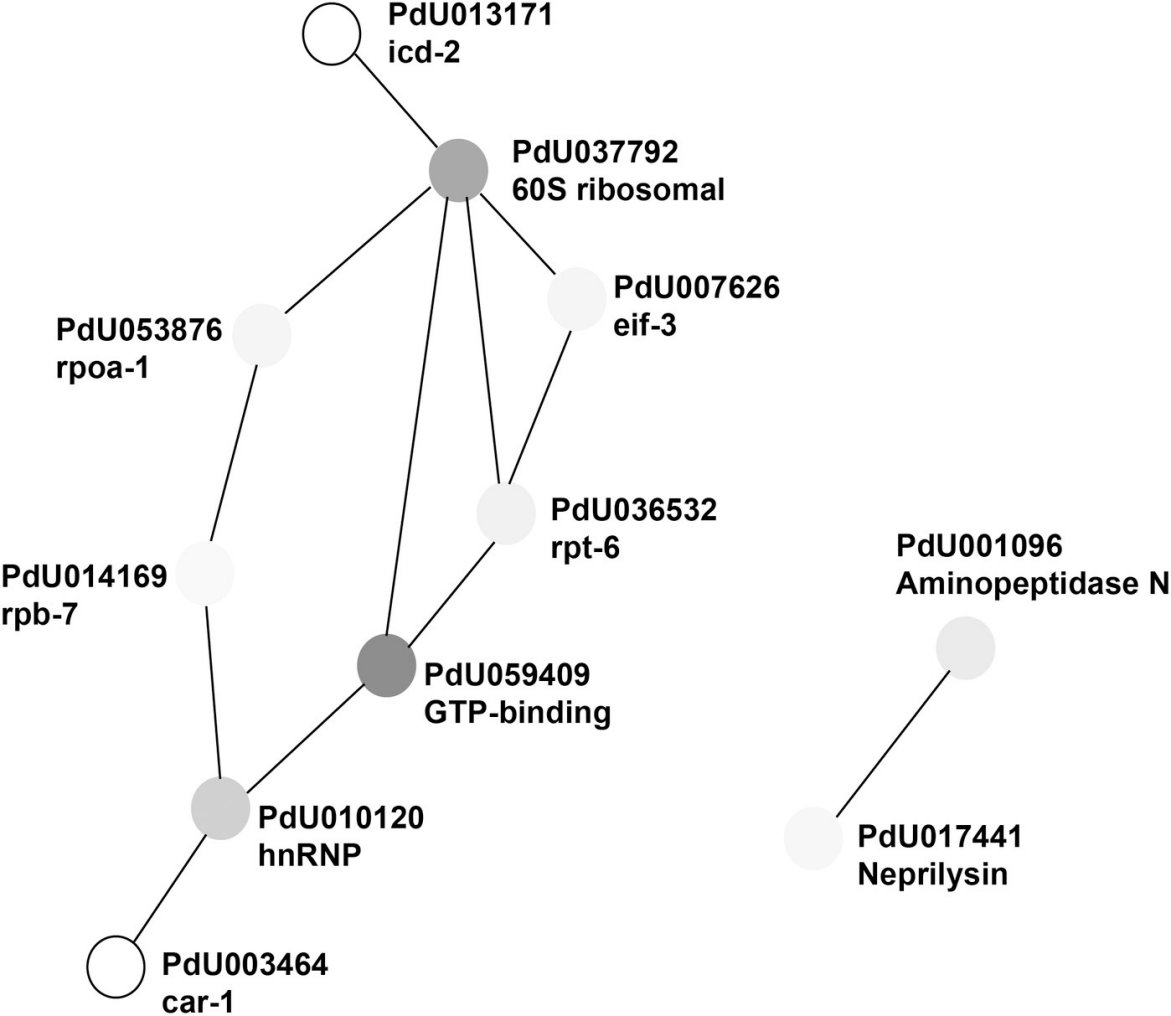
Depiction of the up-regulated long-term freezing protein matches that were grouped together into a functional network through STRING [34]. Matches from Table 2 that were disconnected from any other proteins were not depicted. Shading of the nodes is representative of the relative log fold change of the respective protein from Table 2, while being tied to the scale generated from the values of both Tables 1 & 2.

Where the former analysis failed to annotate certain matches, further use of the NCBI BLAST server was used (blast.ncbi.nlm.nih.gov/Blast.cgi), and in some unresolved cases, Motif (www.genome.jp/tools/motif/). The protein network analysis tool, STRING [34], was then used to analyse the differentially expressed protein sets (Tables 1 & 2 and S1 & S2 Tables) in terms of enriched GO, KEGG, Pfam and other categories. Proteins networked through interaction are shown in Figs 1 and 2 (only those upregulated in the short and long term freezing treatments), and indicated in Tables 1 & 2 and S1 & S2 Tables. S5 to S8 Tables (for the respective treatments and differentiated by whether the sets are up- or down-regulated) show the resulting enriched categories determined through STRING when applicable.

## Results & discussion

### Gene set enrichment analysis

S4 Fig shows biological process GO terms enriched during short term freezing, in which *P*. sp. DAW1 is brought down rapidly to -10ºC and ice nucleated. The largest, top-left network contain GO terms pertaining to structural processes such as cell-cycle, division, stem-cell proliferation and shape, as well as water homeostasis. This network appears to have an analogous cellular component network (S5 Fig) consisting of microtubule, pericentriolar and spindle components. There are also seemingly related processes that are not part of this network that were enriched such as cellular component organization or biogenesis, cell division, endocytosis, and cell proliferation. The other large network depicted in S4 Fig (top-right) consists of nucleotide/nucleoside and DNA metabolism related components, particularly pyrimidines, and translational initiation. This suggests changes to processes such as DNA replication that would accompany the changes in cell-cycle, division, and proliferation processes found in the first network, and possibly DNA methylation mechanisms processing the pyrimidine cytosine. A third smaller network in S4 Fig contains response processes to various ions, potentially due to increased solute concentrations with the loss of liquid water during freezing, or other adaptive processes specific to these ions. A separate two-member network also contains manganese ion transport, and calcium ion import. S6 Fig shows the enriched molecular function terms for short-term freezing, where a network pertaining to ion transport activity can be seen, similar to the small network discussed from the biological processes of S4 Fig. There are also enriched functions relating to DNA and translation processes such as helicase activity, translation factor RNA binding activity, and translation initiation factor binding. S7 Fig, depicting the GO biological processes for the long term freezing treatment, indicates that there are less enriched processes than in short term freezing (S4 Fig). There are two prominent networks in this figure, the top-left involving developmental and morphogenic processes, and the top-right involving translational initiation and spliceosomal complex assembly. Similar to short term freezing, there are also processes involved in various ion responses and metabolism. There are also similarities in the molecular functions, as seen in S9 Fig, where multiple ion binding functions are enriched, as well as translation related functions. S10–S15 Figs depict, in the same order as S4–S9 Figs, the relationship of the enriched categories as treemaps [32].

### Differential expression between treatments

The previous RNA expression profiling study [8] indicated that while there was less transcriptional activity once the nematodes were frozen, there was clear activity nonetheless. The current proteomic analysis mirrors those findings where a wide array of differing functions can be seen both immediately after freezing (Table 1 and S1 Table and, in terms of categories, S5 & S6 Tables), as well as after being frozen for 24 h (Table 2 and S2 Table, and S7 & S8 Tables). Clearly, the two experimental conditions have differing protein expression profiles given that only a small number of up- and down- regulated genes are common between the conditions. When compared to the control (cold acclimation at +5ºC for 3 days brought down from +20ºC), the number of up-regulated proteins when the nematodes were frozen at -10ºC was 47, while the number when held at -10ºC for 24 h was 54. The number of proteins down-regulated at -10ºC compared to the control was 29, and when held at -10ºC for 24 h, 28. By abundance, most notable are the ribosomal proteins. Also strongly represented are genes associated with antioxidation; antiviral, immune and stress response; cytoskeletal and muscle function; proteases; and lipid related activity.

### Upregulated proteins: -10ºC vs control (+5ºC)

Table 1 lists the proteins up-regulated when frozen and ice nucleated at -10ºC, 16 of which are shared with the treatment where the nematodes are held at -10ºC for 24 h. The table also indicates which proteins are linked by interaction in STRING, which was able to incorporate 32 of the 47 genes through its annotation. The largest STRING network consists of 18 proteins (see Fig 1), dominated by the high level GO categories of ion and cyclic binding. In addition, a well represented Molecular Function category, also of a high level, was catalytic activity. The full listing can be seen in S5 Table.

A scan of Table 1 shows a wide variety of other functions: Immune, antiviral and apoptotic function, among other stress-related proteins; muscle and motility; lipid interaction; thermogenesis and thermoregulation; and oxidoreductase. Among the specific proteins listed in Table 1 is a C-type lectin and a transthyretin-like protein. A C-lectin is interesting in this context even if the function is unclear, because it belongs to the family from which antifreeze proteins are derived [35,36]. In the previous intracellular freezing RNA profiling paper [8], a transthyretin-like gene showed an increase immediately after freezing, declining again after 24 h. This gene was also found to be slightly elevated when frozen at -10ºC, but downregulated after 24 h when examined through qPCR. Apart from being co-regulated with neprilysin [37], in a search for ice active proteins responsible for ice recrystallization inhibition (IRI), it was a transthyretin-like protein that was singled out as a likely candidate, yet was unable to be isolated (David Wharton, per. comm.). This is the second validation of this gene showing upregulation during the moment of freezing [8] yet downregulated after long term freezing, and if it is indeed a form of ice active protein, it is intriguing to think that it might play a role as an ice nucleating protein.

### Upregulated proteins: -10ºC and held for 24 h vs control (+5ºC)

Table 2 lists the proteins upregulated when *P*. sp. DAW1 was frozen at -10ºC, ice nucleated and held for 24 h. The network analysis was able to incorporate 31 of the 54 proteins through its annotation with the *C*. *elegans* database. The largest protein interaction network in this set consists of 9 proteins (Fig 2 and indicated in Table 2). Beside the terms that were reflected in the previous treatment, the largest Gene Ontology categories relate to metabolic processes. Cellular Component categories that are enriched include both the intracellular and the cytoplasm, as was also found in the short term freezing treatments. See S7 Table for the full list.

Analysis on the transcriptome [7] showed that genes involved in protein metabolism represent the largest functional grouping, and previous RNA-seq work found that after 24 h of being frozen, protease expression was the most abundant cluster of transcripts [8]. We see a similar response in the protein expression. As has been previously postulated, this may be immune related, or it could be due to energy production coupled with protein metabolism. Although we have no evidence of what pathways are responsible for energy production while frozen, even at a basal level, it may be that protein catabolism is a source. Protease activity at the very least may be part of a metabolic reorganization where unnecessary proteins are degraded.

The two zinc metalloproteases, aminopeptidase N and neprilysin-1 (joined together in Fig 2), were upregulated after 24 h, just as in the previous RNA-seq study. Neprilysin is the most intriguing gene to have emerged from either the previous RNA expression study or the current proteomics study. That it has now been found strongly regulated during intracellular freezing states during RNA expression, where there was an abundance of differing neprilysin-like transcripts, qPCR, and protein expression, lends an urgency to understanding its role. Although previously discussed as to its wide ranging functions [8], recently Fazekas *et al*. [38] have found neprilysin to be one of only four upregulated genes expressed in a 28-year old frozen carcinoma cell line kept in liquid nitrogen when compared to an equivalent, but commercially available, culture.

After being frozen for 24 h, one can see that a late embyogenic abundant (LEA) gene is expressed as well as a leucine rich repeat, both of which were found previously through qPCR [8]. The significance of the LEA proteins as a protection from protein denaturing is now well established [39], while the leucine rich repeat (lrr), in large part owing to its repetitive structure, has been implicated in ice recrystallisation [40]. Annotation of different lrr genes shows up in both treatments (through soc-2 and the ZU5 and death domains transcripts).

### Downregulated proteins: -10ºC vs control (+5ºC)

19 of the 29 genes were included in the network analysis, with only one resulting interaction network of 3 proteins, and few functional enrichments. Among the full list of downregulated proteins in S1 Table is the inclusion titin and α-tubulin, as well as the antioxidant proteins cytosolic glutathione S-transferase 2, two glutathione 2-transferases and superoxide dismutase. See S6 Table for the STRING functional enrichments.

### Downregulated proteins: -10ºC and held for 24 h vs control (+5ºC)

16 out of 28 protein sequences were annotated in the network analysis, with only 4 muscle related proteins forming the largest interaction network (indicated among the listing of downregulated proteins in S2 Table).

Downregulation of multiple muscle, movement and cytoskeletal related proteins is one of the stronger general signals among the listed proteins with the titin and α-tubulin downregulated in short-term freezing and myosin tail, troponin C, dynein light chain 2B, plectin, and spectrin alpha chain downregulated in long-term freezing.These may be tied closely with previous results showing upregulation of collagen/cuticle RNA [8]. But while the previous results of increased collagen at the early onset of cooling indicates possible water distribution, changes in cellular and whole body morphology after freezing would more likely preference less need for energy by not utilizing muscle machinery.

Antioxidant (AO) proteins follow a similar pattern to the muscle and cytoskeletal machinery, where short term freezing has led to a downregulation in cytosolic glutathione S-transferase 2, glutathione 2-transferase and superoxide dismutase and, after 24 h, cytosolic glutathione S-transferase 2. One implication is that the genes combating reactive oxygen species (ROS) are more highly expressed at the stage where the nematodes are being acclimated at +5 (this would presumably also be the case during thawing when oxidative stress would also be expected to be high). In higher organisms, it has previously been hypothesised that some animals may prepare for ROS stress by pre-emptively increasing AO enzymes or activity [41,42].

This possible antioxidant response mirrors what we have come to understand about the way the sugar trehalose is also potentially accumulated. Trehalose is used as a cryoprotectant in *P* sp. DAW1, but an acclimation period is necessary for its production [43], when transcripts associated with trehalose are highly upregulated. The results here further support the idea that trehalose biosynthesis may only be occurring during this period [8,17], given the lack of any genes associated with its production. Such a prepared accumulation of cryoprotectant would not be unique [44,45], and in fact seems an optimal strategy, adapted to prepare for whatever environmental perturbation is necessary, whether a rapid temperature drop (intracellular freezing), or a slower one (cryoprotective dehydration).

## Conclusion

The proteomic picture presents a complex assortment of functions as would be expected of a system undergoing such an extreme disruption. Among other processes, we see downregulation of antioxidant proteins and the machinery related to muscle and the cytoskeleton. Some immune and stress responses, however, are upregulated, as are proteases, which may well indicate energy from catabolism. Such an increase of components of protein synthesis and degradation may also indicate protein cold denaturation or difficulties in synthesising properly-folded proteins at low temperatures.Arguably, just as important are genes that were not highlighted in the analysis. For example, there is no detectable aquaporin, even though previous qPCR analysis indicates a role during freezing[8]. The absence of other potentially important proteins may well be an indication of such constitutive expression, in a similar manner to trehalose. In the case of aquaporin, if constitutive, it might well be that its expression is in aid of its potential role in cryoprotective dehydration, rather than the more abrupt response needed for intracellular freezing.

After having undertaken a number of exploratory and functional approaches (transcriptome and genome [7], qPCR [8,17], RNAi [9], RNA expression [8], proteomics) toward understanding the molecular basis of intracellular freezing, Table 3 provides a partial summary of specific proteins that have been considered as particularly relevant in their respective roles for intracellular freezing survival.

**Table 3:** A partial listing (and the methodological source of evidence for upregulation during short or long term freezing–indicated by an x) of specific genes and their potential role (or lack) in the survival of intracellular freezing. More general and complicated processes (in terms of their signals), such as cytoskeletal genes and antioxidants, are not included.

Proteins	qPCR [8]	RNA expression [8]	Protein expression	Description
Neprilysin	x	x	x	This insulin degrading enzyme has consistently been highly expressed during freezing. It is unclear what its function in this context is, but very likely to be significant.
Leucine Rich Repeat	x	x	x	Leucine rich repeat genes have been shown to exhibit ice recrystallisation inhibition properties [40].
Transthyretin-like protein		x	x	An amyloid cleaing protein (like neprilysin), that has been considered a candidate ice active protein (D. Wharton, pers comm.).
Late Embryogenic Abundant (LEA)	x		x	The genome contains a number of LEA genes and it is highly expressed during long term freezing, hardly surprising given its role as a chaperone in other cold tolerance studies.
C-type Lectin	x		x	C-type lectins have been expressed in the lead up to and immediately after freezing. This carbohydrate-binding family of proteins is the presumed origin of at least one class of antifreeze proteins [35], but its role in intracellular freezing is unclear.
Trehalose				Along with gob (trehalose-6-phosphate phosphatase) and trehalase, it was not expressed in the proteomic analysis {when compared to the control). But the vital role of trehalose in cold tolerance, the likely duplication of the genes [7], and its expression during acclimation [17]. indicates that it may be expresssed constitutively.
Aquaporin	x			Although expressed through qPCR at all stages of freezing (short and long term), it has not been expressed significantly through other methods. It may well be contitutively expressed (as suggested for trehalose), and possibly only in readiness for cryoprotective dehydration.
Desaturase				Desaturase, providing membrane fluidity, has been expressed as the temperature decreases, but does not seem to play a role after freezing.

However, despite all efforts, there continues to be no clear sign of which proteins may be involved in ice recrystallisation inhibition (IRI). Such an ice active protein could of course be one with a dual or even of multiple functions or, as discussed previously [8], it could be a glycolipid [46] or some other product. Given that there is no thermal hysteresis in P sp DAW1 [18], nor any reason for it (since at rapid rates, it would be desirable for ice nucleation to occur intracellularly), then any IRI product may in fact be too minute to be detected with techniques attempted thus far. The search for the ice active proteins, if indeed proteins are responsible, remains.

## Supporting information

S1 File (FASTA)

S1 Fig A gel stained with Coomassie Brilliant Blue. Each gel lane was cut in 5 bands. 2M is the control, 5F, short term freezing, and 6, long-term freezing.
(PDF)

S2 Fig Volcano plot of differential protein expression between control and short term freezing (brought down to -10 and ice nucleated) conditions. Proteins of interest are highlighted in blue (up-regulated) or red (down-regulated). Criteria for proteins of interest are ±2-fold changes (± 1 log2(fold change)) and *p*value < 0.05. A few of the blue and red proteins shown were found to be contaminants and were not included in Table 1 & S1 Table.
(PDF)

S3 Fig Volcano plot of differential protein expression between control and long term freezing (held at -10 for 24 hours) conditions. Proteins of interest are highlighted in blue (up-regulated) or red (down-regulated). Criteria for proteins of interest are ±2-fold changes (± log2(fold change)) and *p*-value < 0.05. A few of the blue and red proteins shown were found to be contaminants and were not included in Table 2 & S2 Table.
(PDF)

S4 Fig Enriched GO term networks of biological processes for short term freezing. The legend describing the relative p-value for each category is also used for S5–S9 Figs.
(PDF)

S5 Fig Enriched GO term networks of cellular components for short term freezing. (PDF)

S6 Fig Enriched GO term networks of molecular functions for short term freezing. (PDF)

S7 Fig Enriched GO term networks of biological processes for long term freezing. (PDF)

S8 Fig Enriched GO term networks of cellular components for long term freezing. (PDF)

S9 Fig Enriched GO term networks of molecular functions for long term freezing. (PDF)

S10 Fig Enriched GO term tree map of biological processes for short term freezing. (PDF)

S11 Fig Enriched GO term tree map of cellular components for short term freezing. (PDF)

S12 Fig Enriched GO term tree map of molecular functions for short term freezing. (PDF)

S13 Fig Enriched GO term tree map of biological processes for long term freezing. (PDF)

S14 Fig Enriched GO term tree map of cellular components for long term freezing. (PDF)

S15 Fig Enriched GO term tree map of molecular functions for long term freezing. (PDF)

S1 Table (XLSX)

S2 Table (XLSX)

S3 Table (XLSX)

S4 Table (XLSX)

S5 Table (XLSX)

S6 Table (XLSX)

S7 Table (XLSX)

S8 Table (XLSX)
