# Correction: Proteomics of intracellular freezing survival

**DOI:** 10.1371/journal.pone.0239641

**Published:** 2020-09-17

**Authors:** Michael A. S. Thorne, Nina Kočevar Britovšek, Liam Hawkins, Kathryn S. Lilley, Kenneth B. Storey

The fifth author's name is spelled incorrectly. The correct name is: Kenneth B. Storey. The correct citation is: Thorne MAS, Britovšek NK, Hawkins L, Lilley KS, Storey KB (2020) Proteomics of intracellular freezing survival. PLoS ONE 15(5): e0233048. https://doi.org/10.1371/journal.pone.0233048
