# Supplementary material for: Proteomics of intracellular freezing survival
Source: PLoS One. 2020 May 26;15(5):e0233048. doi: 10.1371/journal.pone.0233048 (PMC7250440; doi:10.1371/journal.pone.0233048)
Supplement: S1 Fig — Each gel lane was cut in 5 bands. 2M is the control, 5F, short term freezing, and 6, long-term freezing. (PDF) [file pone.0233048.s002.pdf]

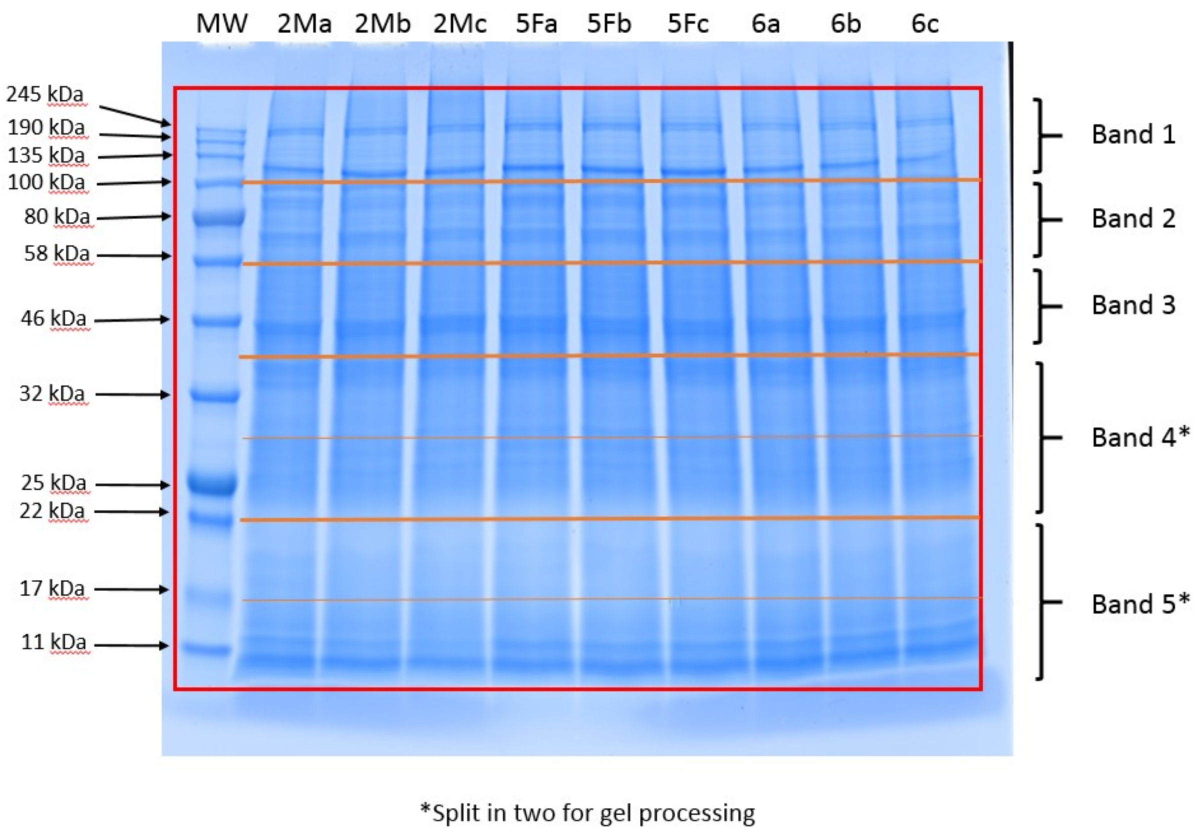

Supplementary Figure 1. A gel stained with Coomassie Brilliant Blue. Each gel lane was cut in 5 bands. 2M is the control, 5F, short term freezing, and 6, long-term freezing.
