# Supplementary material for: Proteomics of intracellular freezing survival
Source: PLoS One. 2020 May 26;15(5):e0233048. doi: 10.1371/journal.pone.0233048 (PMC7250440; doi:10.1371/journal.pone.0233048)
Supplement: S2 Fig — Proteins of interest are highlighted in blue (up-regulated) or red (down-regulated). Criteria for proteins of interest are ±2-fold changes (± 1 log2(fold change)) and pvalue < 0.05. A few of the blue and red proteins shown were found to be contaminants and were not included in Table 1 & S1 Table. (PDF) [file pone.0233048.s003.pdf]

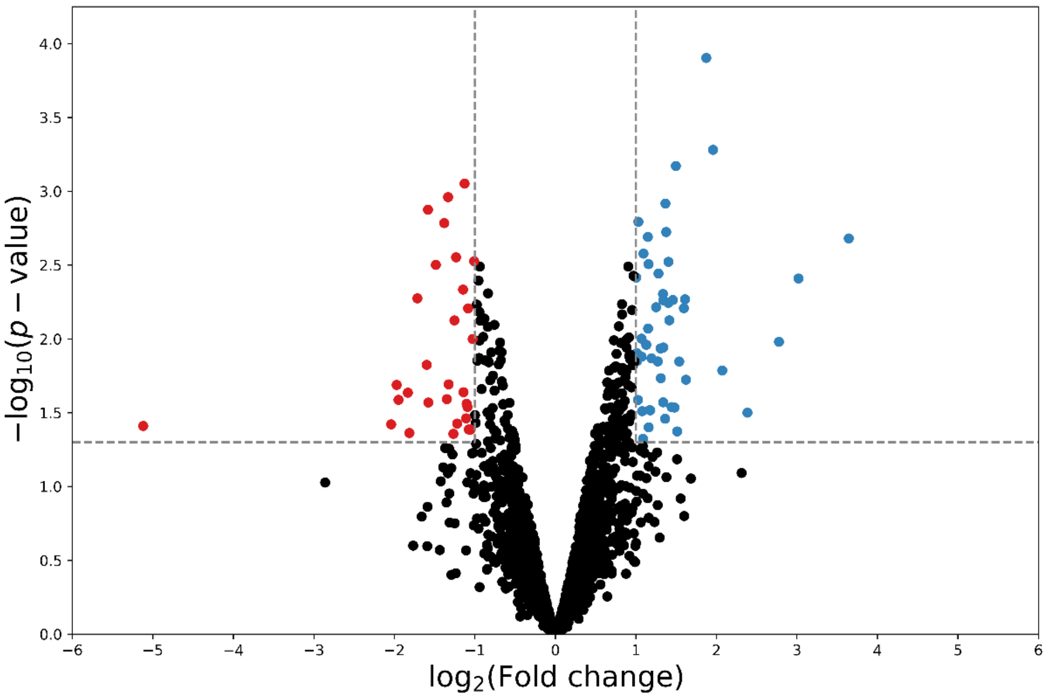

Supplementary Figure 2. Volcano plot of differential protein expression between control and short term freezing (brought down to -10 and ice nucleated) conditions. Proteins of interest are highlighted in blue (up-regulated) or red (down-regulated). Criteria for proteins of interest are ±2-fold changes (± 1 $\log_2$(fold change)) and *p*-value < 0.05. A few of the blue and red proteins shown were found to be contaminants and were not included in Table 1 & Supplementary Table 1.
