# Supplementary material for: Proteomics of intracellular freezing survival
Source: PLoS One. 2020 May 26;15(5):e0233048. doi: 10.1371/journal.pone.0233048 (PMC7250440; doi:10.1371/journal.pone.0233048)
Supplement: S3 Fig — Proteins of interest are highlighted in blue (up-regulated) or red (down-regulated). Criteria for proteins of interest are ±2-fold changes (± log2(fold change)) and p-value < 0.05. A few of the blue and red proteins shown were found to be contaminants and were not included in Table 2 & S2 Table. (PDF) [file pone.0233048.s004.pdf]

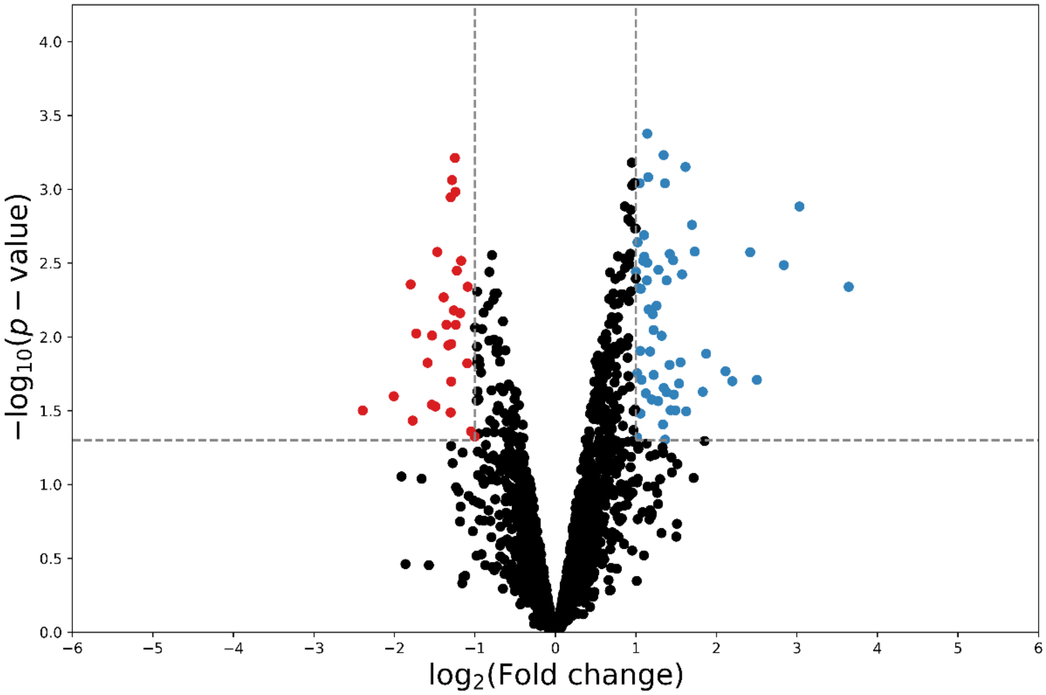

Supplementary Figure 3 Volcano plot of differential protein expression between control and long term freezing (held at -10 for 24 hours) conditions. Proteins of interest are highlighted in blue (up-regulated) or red (down-regulated). Criteria for proteins of interest are ±2-fold changes (± $\log_2$(fold change)) and p-value < 0.05. A few of the blue and red proteins shown were found to be contaminants and were not included in Table 2 & Supplementary Table 2.
