# Supplementary material for: Proteomics of intracellular freezing survival
Source: PLoS One. 2020 May 26;15(5):e0233048. doi: 10.1371/journal.pone.0233048 (PMC7250440; doi:10.1371/journal.pone.0233048)
Supplement: S4 Fig — The legend describing the relative p-value for each category is also used for S5–S9 Figs. (PDF) [file pone.0233048.s005.pdf]

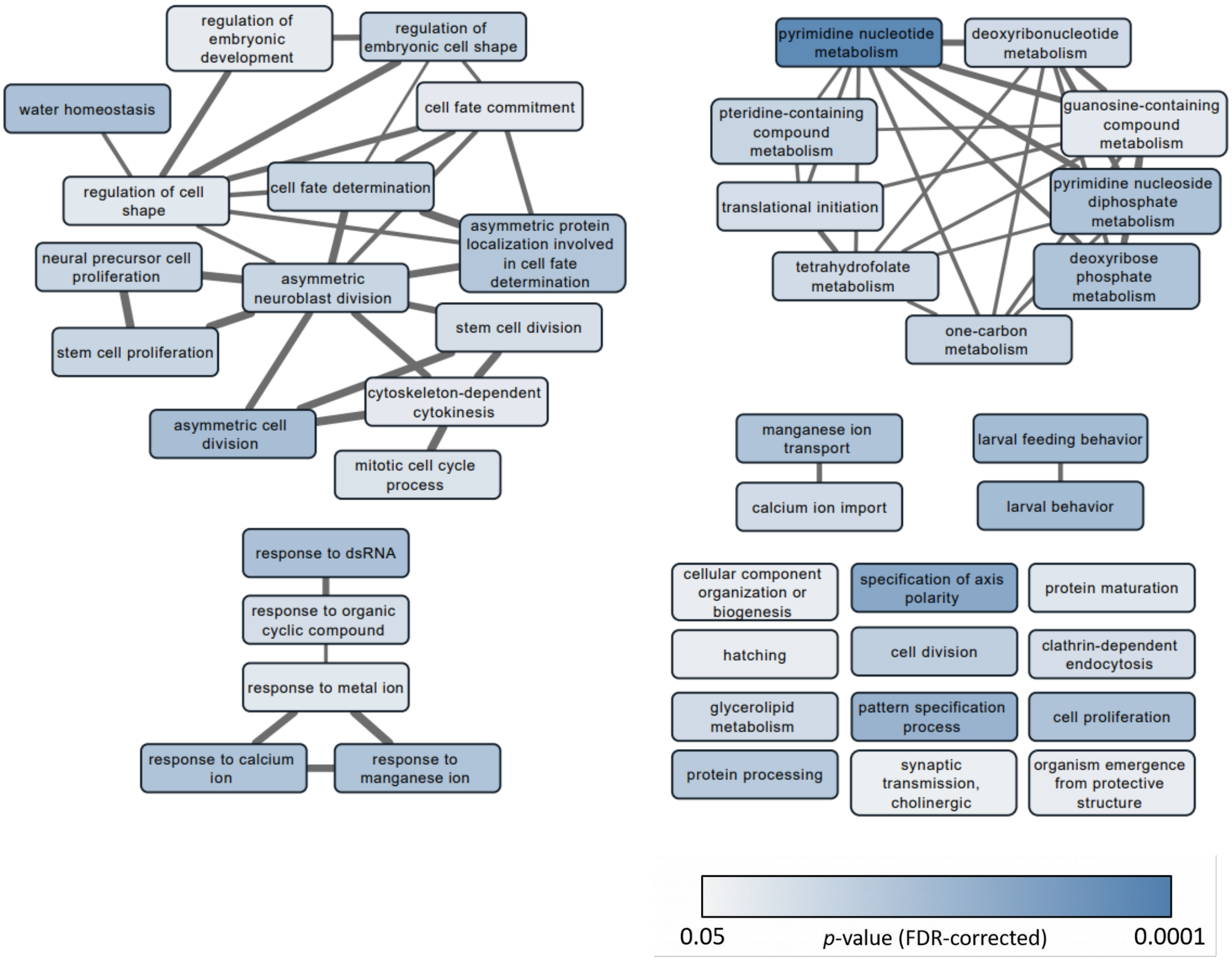

Supplementary Figure 4. Enriched GO term networks of biological processes for short term freezing. The legend describing the relative p-value for each category is also used for Figures 5-9.
