# Supplementary material for: Proteomics of intracellular freezing survival
Source: PLoS One. 2020 May 26;15(5):e0233048. doi: 10.1371/journal.pone.0233048 (PMC7250440; doi:10.1371/journal.pone.0233048)
Supplement: S6 Fig — (PDF) [file pone.0233048.s007.pdf]

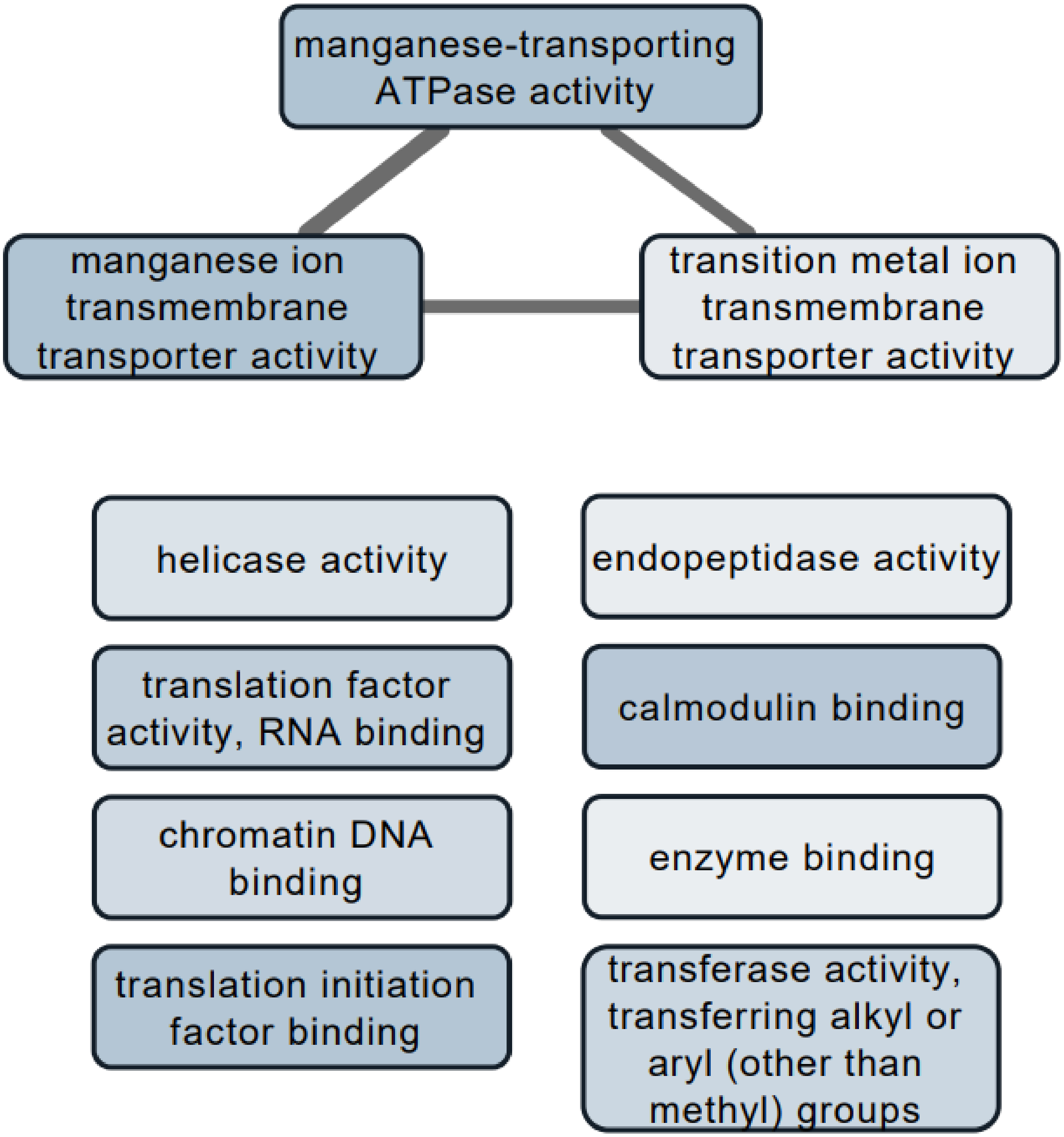

Supplementary Figure 6. Enriched GO term networks of molecular functions for short term freezing.
