# Supplementary material for: Proteomics of intracellular freezing survival
Source: PLoS One. 2020 May 26;15(5):e0233048. doi: 10.1371/journal.pone.0233048 (PMC7250440; doi:10.1371/journal.pone.0233048)
Supplement: S7 Fig — (PDF) [file pone.0233048.s008.pdf]

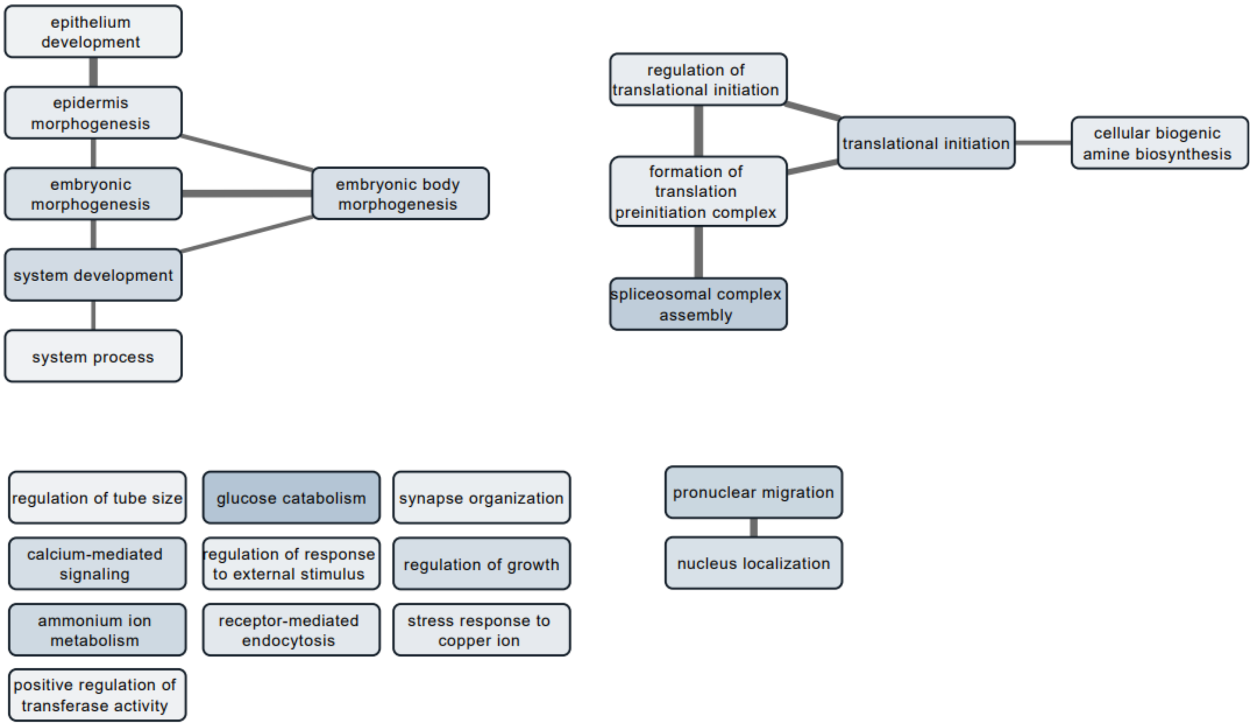

Supplementary Figure 7. Enriched GO term networks of biological processes for long term freezing.
